# Biological Evaluation of ^131^I- and CF750-Labeled Dmab(scFv)-Fc Antibodies for Xenograft Imaging of CD25-Positive Tumors

**DOI:** 10.1155/2014/459676

**Published:** 2014-04-27

**Authors:** Qing Fan, Huawei Cai, Hao Yang, Lin Li, Cen Yuan, Xiaofeng Lu, Lin Wan

**Affiliations:** ^1^Lab of Transplant Engineering and Immunology, Regenerative Medicine Research Center, West China Hospital, Sichuan University, Chengdu 610041, China; ^2^Department of Nuclear Medicine, West China Hospital, Sichuan University, Chengdu 610041, China

## Abstract

A Dmab(scFv)-Fc antibody containing the single chain variable fragment of a humanized daclizumab antibody and the Fc fragment of a human IgG1 antibody was produced via recombinant expression in* Pichia pastoris*. The Dmab(scFv)-Fc antibody forms a dimer in solution, and it specifically binds CD25-positive tumor cells and tumor tissues. For tumor imaging, the Dmab(scFv)-Fc antibody was labeled with the 131I isotope and CF750 fluorescent dye, respectively. After intravenous injection of mice bearing CD25-positive tumor xenografts, tumor uptake of the ^131^I-Dmab(scFv)-Fc antibody was visible at 1 h, and clear images were obtained at 5 h using SPECT/CT. After systemic administration of the CF750-Dmab(scFv)-Fc antibody, tumor uptake was present as early as 1 h, and tumor xenografts could be kinetically imaged within 9 h after injection. These results indicate that the Dmab(scFv)-Fc antibody rapidly and specifically targets CD25-positive tumor cells, suggesting the potential of this antibody as an imaging agent for the diagnosis of lymphomatous-type ATLL.

## 1. Introduction

Adult T-cell leukemia/lymphoma (ATLL) is a rare but aggressive T-cell malignancy that is linked to infection by the retrovirus HTLV-1 [1]. According to the clinical features and prognosis of patients, ATLL is classified into four subtypes: lymphomatous, acute, chronic, and smoldering [2–4]. The lymphomatous and acute subtypes are fast-growing forms of ATLL that have a poor prognosis. Lymphomatous ATLL accounts for 20% of ATLL cases, and it has a median survival of 6 months to 2 years. The acute form of ATLL accounts for 60% of all ATLL cases, and it has a median survival of only 6 months. The chronic and the smoldering subtypes of ATLL are less aggressive, and they have median survival times of 2 and 5 years, respectively [5].

The pathogenesis of ATLL is generally characterized by HTLV-1 infection, lymphoid proliferation, and the formation of lymphoma or leukemia [5]. Due to its diverse clinical features, the subtype-dependent diagnosis of ATLL is usually established using both clinical and laboratory techniques [1, 6]. Serological assays have been used to detect HTLV-1 infection. A blood or bone marrow biopsy is usually used to determine the white blood cell count and the flower-like shape of T cells and to measure calcium levels when diagnosing T cell leukemia [7, 8]. Lymphomatous ATLL is observed primarily in the lymph nodes and the skin, liver, spleen, lung, gastrointestinal tract, bone, and central nerve system frequently display ATLL mediated-lesions. In addition to blood examination, immunohistochemistry of a suspicious lesion is essential for the diagnosis of these diseases [9]. However, histopathological examination is limited by the difficulty of obtaining sufficient tumor tissue [6]. There is an urgent need to develop novel, noninvasive tools for the early diagnosis of ATLL, especially the lymphoma subtype.

In recent years, noninvasive molecular imaging has become popular for the diagnosis of solid tumors [10–12]. Due to their high specificity and affinity for antigens, antibodies against tumor cell-specific surface markers are ideal for tumor imaging [13]. Chemokine receptors mediate the migration and tissue localization of lymphocytes. ATLL-specific receptors include CCR4, CCR7, CCR8, and CXCR4 [14]. In addition, ATLL cells also highly express other surface proteins, including CD25, TSLC1, CD52, CD56, CD70, and cancer/testis antigen [15–20]. Of these surface proteins, CD25 is constitutively expressed on most ATLL cells [14]. Treatment with radionuclide-conjugated antibody against CD25 has led to clinical responses in the majority of patients with ATLL [15, 21]. Consequently, CD25 expression was included in the minimal required panel for ATLL diagnosis [3]. These data suggest that antibodies against CD25 might be developed as imaging tools for ATLL diagnosis.

It is well known that large, intact antibodies are limited in tumor imaging due to their incomplete tumor penetration and slow blood clearance [22]. Consequently, various forms of smaller antibodies have been developed in recent decades [23]. The single chain fragment of the variable (scFv) antibody was first constructed by genetic fusion of the variable regions of the heavy and light chains [24]. However, this monovalent scFv antibody was limited by its low affinity for antigen and low tumor retention caused by rapid blood clearance [23]. Based on the scFv antibody, a spectrum of divalent single-chain antibodies have been developed, including diabodies, minibodies, and scFv-Fc antibodies. These divalent antibodies exhibit higher affinity and tumor uptake compared to the monovalent scFv antibody. In addition, because of their rapid targeting and faster clearance compared to intact antibodies, divalent antibodies are widely used for tumor imaging [13, 23, 25].

Currently, radiolabeled antibodies predominately occupy the field of antibody-based tumor imaging by positron emission computed tomography (PET) and single photon emission computed tomography (SPECT). A variety of radionuclides have been conjugated to antibodies for radioactive imaging [13]. To avoid the risks associated with radiolabeled antibodies, many optical imaging probes have been developed in recent years [26]. Of these probes, near-infrared (NIR) fluorescence probes are the most attractive due to their lower absorption and autofluorescence in normal tissues in the NIR region (700–900 nm) than that of the visible region. Due to their specificity, high sensitivity, low cost, and absence of ionizing radiation, NIR-based optical imaging might become a preferable alternative over radioisotope-based imaging, especially in the experimental setting and image-guided clinical surgery [13].

Daclizumab is a humanized intact antibody with high affinity and specificity for CD25 [15]. We previously synthesized the gene encoding scFv antibody of daclizumab and further developed an Dmab(scFv)-Fc antibody [27]. The specificity and high affinity of the Dmab(scFv)-Fc antibody for CD25 made it attractive for imaging CD25-positive tumors. Here, we radiolabeled the Dmab(scFv)-Fc antibody and investigated its tumor uptake and tissue distribution in mice bearing CD25-positive tumor xenografts. Subsequently, we labeled the Dmab(scFv)-Fc antibody with a NIR fluorescent dye and evaluated the tumor-targeting capability of this antibody using an optical imaging system.

## 2. Materials and Methods

### 2.1. Preparation of the Dmab(scFv)-Fc Antibody

Expression and purification of the Dmab(scFv)-Fc antibody was performed according to our previous work [27], with some modifications. Briefly,* P. pastoris* GS115 cells containing the pPIC 9 K-Dmab(scFv)-Fc plasmid were used to produce the Dmab(scFv)-Fc antibody. Cells derived from a single colony were inoculated into 25 mL buffered glycerol-complex medium (BMGY) and incubated at 28°C with shaking (280 rpm) overnight. Then, the culture was transferred into 1 L fresh BMGY. When the *A*_600 nm_ of the culture reached approximately 5, all the cells were collected by centrifugation at room temperature (3,500 g for 5 min) and resuspended in 100 mL buffered methanol-complex medium (BMMY). To induce production of the Dmab(scFv)-Fc antibody, 3% methanol was added to the media daily. After 72 h of induction, the culture supernatant was collected by centrifugation at 4°C (15,000 g for 15 min). Subsequently, the supernatant was dialyzed against binding buffer (50 mM Tris-HCl, 0.5 M NaCl, and 10 mM imidazole, pH 8.0) at 4°C overnight. Finally, the Dmab(scFv)-Fc antibody was purified using Ni-NTA agarose (Qiagen, CA, USA), according to the manufacturer's protocol. The purified antibody was dialyzed against phosphate-buffered saline (PBS) (8 g  L^−1^  NaCl, 0.2 g L^−1^  KCl, 3.49 g L^−1^  Na_2_HPO_4_ *·*12H_2_O, and 0.2 g L^−1^ KH_2_PO_4_) at 4°C overnight. The protein concentration was measured using the Bradford method. Approximately, 60–70 mg antibody was recovered from 1L supernatant. The monovalent single chain fragment of the variable antibody against CD25 Dmab(scFv) was prepared according to our previous protocol [28].

### 2.2. Size-Exclusion Chromatography

A Superdex 75 10/300 GL column (GE Healthcare, Sweden) and Pure system (AKTA purifier 10, GE Healthcare, Sweden) were used to perform size-exclusion chromatography. The purified antibody (300~360 *μ*g) was loaded onto the column and eluted with PBS at a rate of 0.4 mL/min. The apparent molecular weight of the antibody was calibrated by protein markers, including thyroglobulin (670,000), *γ*-globulin (158,000), bovine serum albumin (67,000), ovalbumin (44,000), myoglobin (17,000), and vitamin B12 (1,350).

### 2.3. Sodium Dodecyl Sulfate Polyacrylamide Gel Electrophoresis

The protocol used for sodium dodecyl sulfate polyacrylamide gel electrophoresis (SDS-PAGE) was described in our previous work [29]. Briefly, the purified antibody (6 *μ*g) was separated on a 10% gel in the presence or absence of 2-mercaptoethanol (2-ME) and visualized by Coomassie Brilliant Blue staining.

### 2.4. Cell Binding Assays

CD25-positive Hut102 cells and CD25-negative SMMC7721 cells were purchased from the American Type Culture Collection (ATCC, VA, USA). Cells were cultured in RPMI 1640 medium supplemented with 10% serum (fetal bovine serum for Hut102 or calf serum for SMMC7721), 2 mmol/L L-glutamine, 100 U/mL penicillin, and 100 *μ*g/mL streptomycin at 37°C in a humidified atmosphere containing 5% CO_2_. For cell-binding assays, antibodies were labeled with fluorescein isothiocyanate (FITC, Sigma, USA). Approximately 2 × 10^5^ cells were incubated with the FITC-labeled antibody at different concentrations in 100 *μ*L PBS containing 0.5% calf serum at 37°C for 1 h in darkness. After two washes with PBS containing 0.5% calf serum, the cells were analyzed using a flow cytometer (Cytomics FC 500, Beckman Coulter, CA, USA). A FITC-labeled isotype antibody was utilized as a control in these assays. The EC50 (amount of antibody for 50% binding) of antibody was calculated according to the perspective binding rate curve.

### 2.5. Immunofluorescence Histochemistry

Tumor and liver tissues derived from mice bearing Hut102 xenografts were paraffin embedded and sectioned. After antigen retrieval, the paraffin sections were incubated with different concentrations of FITC-labeled antibody in the dark at 4°C overnight. The nuclei were labeled by DAPI staining, and the sections were examined using a fluorescence microscope (Leica DM 4000B, Germany). A FITC-labeled isotype antibody was utilized as a negative control.

### 2.6. Tumor Xenograft Animal Model

All of the protocols used in this report were approved by the University Animal Care and Use Committee. Female BALB/C nu/nu mice (4–6 weeks) were injected intraperitoneally with a single dose of cyclophosphamide (10 mg/kg). Two days later, approximately 1 × 10^6^ Hut102 cells were implanted subcutaneously at the postauricular region of the mouse. Tumor growth was monitored every day, and tumor volumes were calculated according to the following formula: width^2^ × length × 0.5. The colon cancer cells (LS174T) were used as a CD25-negative tumor control. To produce a dual tumor grafts model, LS174T cells and Hut102 cells were simultaneously implanted in the left and right hind legs, respectively.

### 2.7. Labeling the Antibody with Radionuclide

^131^I-labeled sodium iodide (China Isotope & Radiation Co. Ltd, Chengdu, China) was used in this experiment. Briefly, 50 *μ*g antibody (1 mg/mL) was transferred into a clean Eppendorf tube. Subsequently, 7.4 MBq of ^131^I-labeled sodium iodide (specific radioactivity ≥ 3.7 MBq/*μ*L) and 10 *μ*g of N-bromosuccinimide (1 mg/mL) were added to the antibody, and this mixture was incubated at room temperature for 5 min with occasional shaking. Free sodium iodide was removed using a PB-10 desalting column with PBS as the running buffer. The degree of labeling (DOL) was analyzed by thin layer chromatography (TLC). Total radioactivity of labeled antibody was determined using a FJ-2008PS Gamma counter (Xi'an Nuclear Instrument Factory, Shanxi, China). The specific activity was expressed as radioactivity per milligram antibody. Immunoreactivity of ^131^I-labeled antibody was analyzed by dose-dependent cell binding assays. The mixture of antibody and free ^131^I was used as control.

### 2.8. Single Photon Emission Computed Tomography Imaging of Mice Bearing Xenograft Tumors

Single photon emission computed tomography (SPECT) imaging of mice bearing tumor xenografts was performed using a Precedence 6 slice SPECT/CT (Philips Medical Systems, Milpitas, CA, USA). To reduce the background of the thyroid, the mice were fed with water containing 0.1% sodium iodide for one week prior to imaging. After intravenous injection of ^131^I-labeled antibody (185 kBq/g body weight), three mice were anesthetized with 2% isoflurane and scanned at 1, 3, 5, 9, and 24 h, respectively. Helical SPECT images were acquired in 30 projections over 15min using a double-headed camera. CT images were acquired in 30 projections with a 1000ms exposure time using a 45kVP X-ray source over 5min. Whole-body radionuclide images were reconstructed using an iterative ordered subset expectation maximization two-dimensional algorithm, and these images were fused with CT images using Syntegra software (Philips Medical Systems, Milpitas, CA). Regions of interest (ROIs) were drawn in all planes over the tumor, liver, and muscle region on the flank opposite of the tumor. ROI activities were used to calculate the ratio of tumor to normal tissue.

### 2.9. Biodistribution of the Radiolabeled Antibody in Mice Bearing Xenograft Tumors

After intravenous injection of ^131^I-labeled antibody, three mice were sacrificed at 1, 3, 5, 9, and 24 h after injection, respectively. The organs/tissues of interest were harvested, weighed, and counted for radioactivity in a Gamma counter. The results are expressed as the percent injected dose per gram of tissue (%ID/g).

### 2.10. Labeling the Antibody with CF750, Succinimidyl Ester

The purified antibody was labeled with CF750, succinimidyl ester (CF750, Sigma, CA, USA), according to the manufacturer's instructions. Briefly, CF750 was dissolved in dimethyl sulfoxide (DMSO) to a final concentration of 10 mM. The pH of the antibody solution (1 mg/mL) was adjusted to 8.3 using sodium bicarbonate. CF750 dye was added to the antibody solution at a 12 : 1 molar ratio of dye to antibody. After incubation at room temperature for 1 h, the mixture was dialyzed against PBS with several changes until the *A*_755 nm_ of the dialysate was below 0.01. The degree of labeling (DOL) was calculated according to the following formula: DOL =  (*A*_755 nm_× molecular weight of antibody × dilution factor)/(*ε* × concentration of antibody). The dilution factor was defined in determination of antibody concentration (mg/mL). The molar extinction coefficient (*ε*) of CF750 is 250,000.

### 2.11. Optical Imaging of Mice Bearing Xenograft Tumors

When the tumor volume reached 0.1-0.2 cm^3^, the mice with single (Hut102) or dual (Hut102 and LS174T) tumor grafts were intravenously injected with 100 *μ*L CF750-labeled antibody solution. Subsequently, the injected mice were imaged using inan IVIS optical imaging system (Caliper Life Sciences, CA, USA) at different time points. Finally, the mice were sacrificed, and the uptake of CF750-labeled antibody was analyzed in different organs/tissues. In the control group, the mice were injected with the same amount of unconjugated CF750 dye.

## 3. Results

### 3.1. Antigen Binding Activity and Specificity of the Dmab(scFv)-Fc Antibody

**Figure 1:**
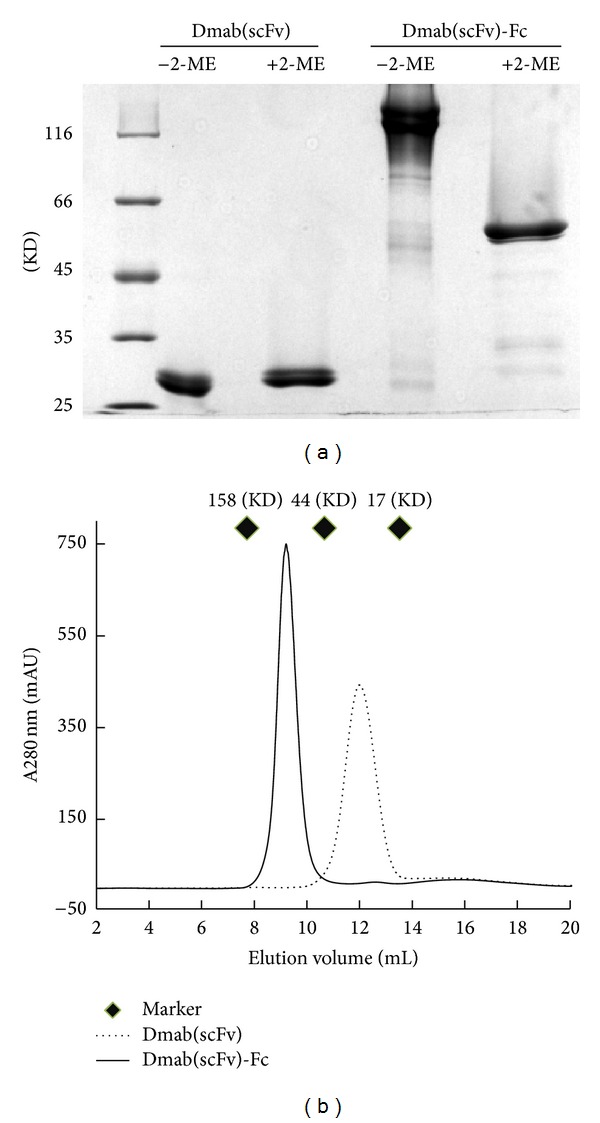
Purification of the Dmab(scFv)-Fc antibody. (a) SDS-PAGE analysis of the Dmab(scFv)-Fc antibody (6 *μ*g) in the presence (+) or absence (−) of 2-ME. (b) Gel filtration chromatography of the Dmab(scFv)-Fc antibody (360 *μ*g). The Dmab(scFv) antibody (300 *μ*g) was used as a control.

As shown in (Figure 1(a)), SDS-PAGE demonstrated that the apparent molecular weight of the Dmab(scFv)-Fc antibody was approximately 120 KD in the absence of 2-ME, whereas it was 60 KD in the presence of 2-ME. In contrast, the apparent molecular weight of the Dmab(scFv) antibody in the absence of ME was identical to that of Dmab(scFv) in the presence of 2-ME. The retention volumes of Dmab(scFv)-Fc and Dmab(scFv) on the Superdex 75 column were approximately 9.19 mL and 11.81 mL, corresponding to the apparent molecular weights of 120 KD and 30 KD, respectively, (Figure 1(b)). These results suggest that Dmab(scFv) exists as a monomer in solution, whereas Dmab(scFv)-Fc forms a dimer due to the incorporation of the Fc domain.

**Figure 2:**
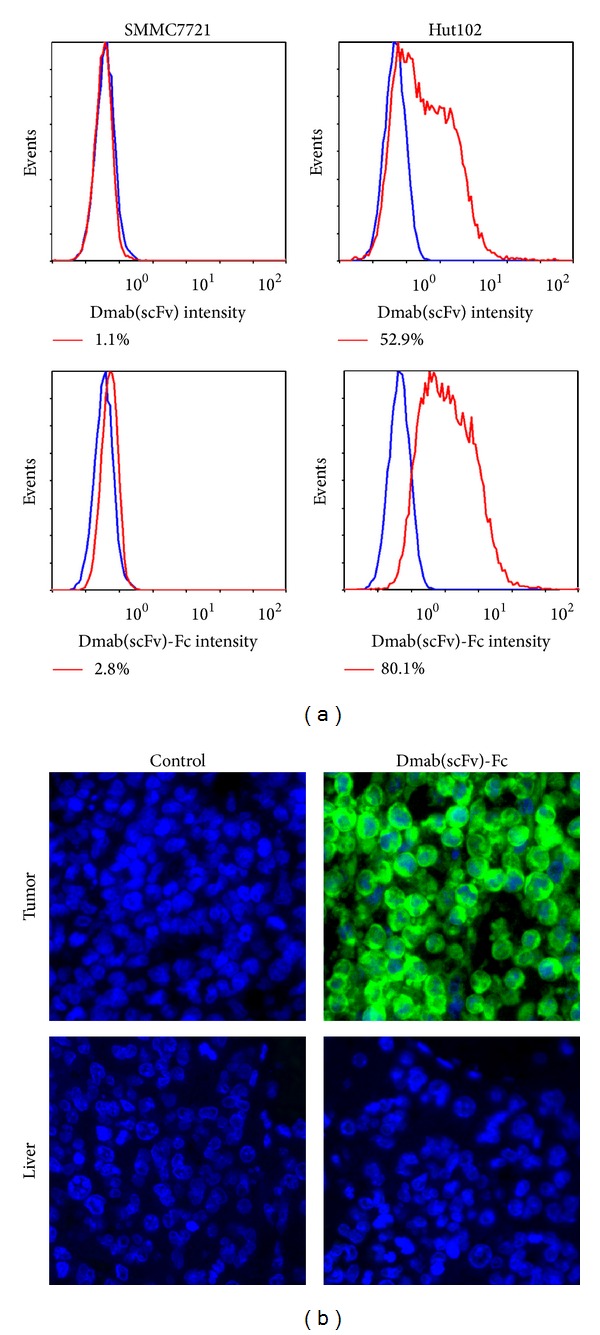
Binding specificity of the Dmab(scFv)-Fc antibody. (a) CD25-negative SMMC7721 cells and CD25-positive Hut102 cells were incubated with FITC-labeled Dmab(scFv)-Fc antibody or Dmab(scFv) antibody, followed by flow cytometric analysis. (b) Hut102 tumor tissues and liver tissues were stained with FITC-labeled Dmab(scFv)-Fc antibody and observed under a fluorescence microscope. DAPI was used to visualize the cell nucleus. An isotype antibody was used as a control.

**Figure 3:**
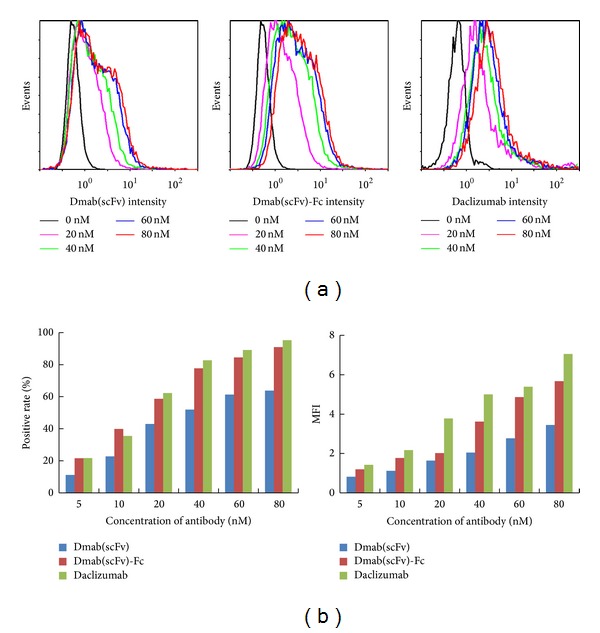
Comparison of the binding ability of the Dmab(scFv) antibody, Dmab(scFv)-Fc antibody, and parental antibody daclizumab. Hut102 cells were incubated with the FITC-labeled antibodies at indicated molar concentration, followed by flow cytometric analysis. The positive rate and MFI of the antibodies were compared.

CD25-positive Hut102 cells and CD25-negative SMMC7721 cells were used to determine the* in vitro* binding ability and specificity of the Dmab(scFv)-Fc antibody. After incubation with the antibody, the binding rate of the Dmab(scFv)-Fc antibody was 80.1% in Hut102 cells and 2.8% in SMMC7721 cells (Figure 2(a)). Further immunohistochemical analyses demonstrated that the Dmab(scFv)-Fc antibody bound Hut102 tumor tissue but not liver tissue (Figure 2(b)). These results indicate that the scFv-Fc antibody specifically binds CD25-positive Hut102 cells. In addition, binding of the Dmab(scFv)-Fc antibody to Hut102 cells is dose-dependent (Figure 3). The binding rates of the Dmab(scFv)-Fc antibody at 5, 10, 20, 40, 60, and 80 nM are 21.6%, 39.9%, 58.7%, 77.7%, 84.5%, and 90.9%, respectively, compared to 11.2%, 22.8%, 43%, 51.9%, 61.3%, and 63.8% for the Dmab(scFv) antibody at the same molar concentration. The mean fluorescence intensity (MFI) of Dmab(scFv)-Fc antibody-stained cells was higher than that of cells stained with the Dmab(scFv) antibody at the same molar concentration. The binding rate and MFI of Dmab(scFv)-Fc antibody are similar to that of the parental antibody daclizumab at the same molar concentration. The EC50 values (amount of antibody for 50% binding) of Dmab(scFv), Dmab(scFv)-Fc, and daclizumab were approximately 36 nM, 17 nM, and 15 nM, respectively. These results suggest that the affinity for CD25 of the divalent Dmab(scFv)-Fc antibody was higher than that of the monovalent Dmab(scFv) antibody.

### 3.2. Biodistribution of the ^131^I-Labeled Dmab(scFv)-Fc Antibody and SPECT/CT Imaging

**Figure 4:**
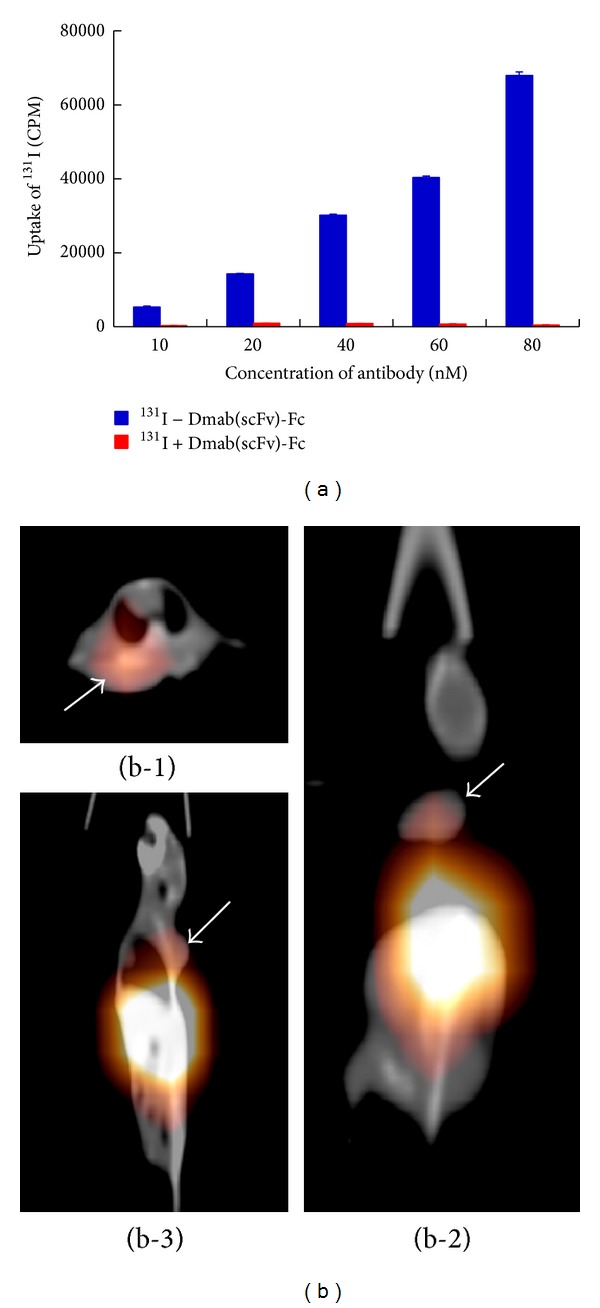
Immunoreactivity of ^131^I-Dmab(scFv)-Fc antibody and SPECT/CT of mice bearing Hut102 tumor xenografts at 5 h after injection. (a) Immunoreactivity of ^131^I-labeled Dmab(scFv)-Fc antibody was analyzed using Hut102 cell binding assays. The same amount of free ^131^I was used as a control. (b) After intravenous injection of ^131^I-labeled Dmab(scFv)-Fc antibody (185 kBq/g body weight), the mice were scanned by using a Precedence 6 slice SPECT/CT machine. Different angles of the tumor graft (arrow) are displayed, including a sectional view (b-1), normal view (b-2), and side view (b-3).

TLC analysis indicated that the radiochemical purity of the ^131^I-Dmab(scFv)-Fc antibody was approximately 92% with specific activity of 37.4 MBq/mg. The ^131^I-Dmab(scFv)-Fc antibody showed dose-dependent binding to Hut102 cells* in vitro* (Figure 4(a)). In Hut102 xenograft model, the mice were intravenously injected with the ^131^I-Dmab(scFv)-Fc antibody when the tumor volume reached 0.4-0.5 cm^3^. Three mice were sacrificed at 1, 3, 5, 9, and 24 h after injection, and the biodistribution of the antibody was analyzed. As shown in Table 1, the ^131^I-Dmab(scFv)-Fc antibody exhibited rapid tumor uptake, with an activity of 28.77 ± 6.43% ID/g at 1 h and 28.94 ± 5.81% ID/g at 3 h. Thereafter, the antibody retention in the tumor decreased over time. However, the activity of the ^131^I-Dmab(scFv)-Fc antibody still persisted at a high level (>13%) in tumors for 5–9 h after injection. As expected, the activity of the ^131^I-Dmab(scFv)-Fc antibody in muscle was significantly lower than that in tumor xenografts. The tumor-to-muscle signal ratios at 1, 3, 5, 9, and 24 h were 2.6 ± 0.64, 2.79 ± 0.38, 4.33 ± 0.94, 4.27 ± 0.85, and 6.44 ± 1.2, respectively (Table 1). Moreover, the lowest accumulation of the ^131^I-Dmab(scFv)-Fc antibody was detected in the brain. The tumor-to-brain ratio increased from 8.56 ± 1.98 at 1 h to 22.42 ± 7.21 at 24 h, which was approximately 4 times higher than the tumor-to-muscle ratio at the same time point. These results indicate that the ^131^I-Dmab(scFv)-Fc antibody specifically localizes to the CD25-positive tumor graft. Whole-body imaging by SPECT/CT further confirmed the tumor-specific targeting of the ^131^I-Dmab(scFv)-Fc antibody. The activity of the ^131^I-Dmab(scFv)-Fc antibody was detectable in the tumor 1 h after injection. Due to the signal reduction in the liver and kidney, a clear image was obtained using SPECT/CT at 5 h after injection (Figure 4(b)). The ROI signal of the ^131^I-Dmab(scFv)-Fc antibody in tumors was two times greater than that in muscle, indicating that the antibody specifically accumulates in tumors.

### 3.3. Tumor Targeting of the CF750-Labeled Dmab(scFv)-Fc Antibody

**Figure 5:**
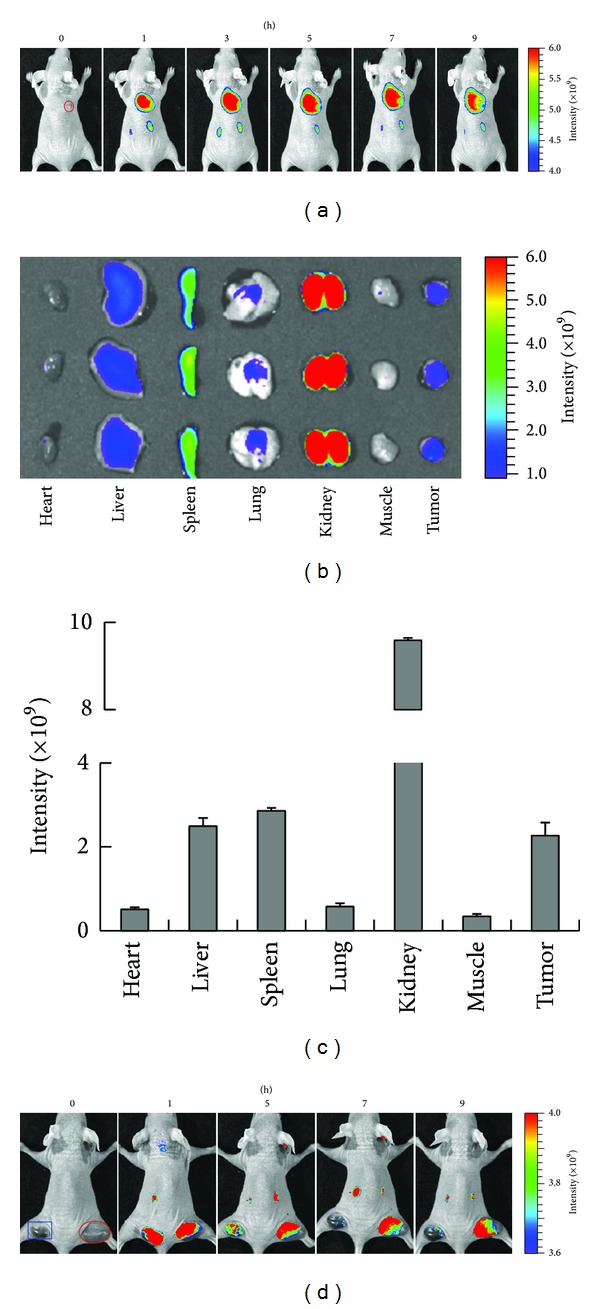
Optical imaging of mice bearing Hut102 tumor xenografts. (a) After intravenous injection of CF750-Dmab(scFv)-Fc antibody, mice (*n* = 3) were anesthetized and scanned at 0, 1, 3, 5, 7, and 9 h, respectively. The Hut102 tumor graft is indicated by a cycle. (b) At 9 h after injection, mice were sacrificed and tissues were collected and scanned. (c) Tissue distribution of the CF750-Dmab(scFv)-Fc antibody in mice at 9 h after injection. (d) CD25-negative LS174T cells and CD25-positive Hut102 cells were implanted in the left (square) and right (cycle) hind legs, respectively. After intravenous injection of CF750-Dmab(scFv), the mice were scanned at 1, 5, 7, and 9 h, respectively.

Tumor targeting of the Dmab(scFv)-Fc antibody was evaluated using an optical molecular imaging system that allows for the kinetic visualization of tumor targeting and antibody clearance in the same animal. The antibody was labeled with CF750, succinimidyl ester. SDS-PAGE analysis demonstrated that the labeling rate was over 80% (data not shown). Under the conditions defined in this experiment, the degree of labeling ranged from 2 to 3, indicating that 2 or 3 dye molecules were conjugated to each antibody molecule. After injection with 100 *μ*g (100 *μ*L) CF750-labeled antibody, mice bearing Hut102 xenograft tumors were scanned by the optical imaging system at different times. As shown in (Figure 5(a)), the xenografts were visible within 1 h after injection, demonstrating the rapid tumor uptake of the CF750-labeled Dmab(scFv)-Fc antibody. Maximum tumor uptake was detected at 3 h, and the signal persisted for 9 h. Although the antibody accumulated in the tumor was still detectable at 48 h, the signal intensity reduced significantly by 24 h (data not shown). To evaluate the biodistribution of the antibody, mice were sacrificed at 9 h, and the signal intensities in several tissues of interest were analyzed. Figures 5(b) and 5(c) revealed that the uptake rate of antibody was as follows (from high to low): kidney > spleen > liver > tumor > lung > heart > muscle. Notably, the antibody uptake in tumor xenografts was comparable to that in the liver at 9 h. The uptake ratios of tumor-to-muscle, tumor-to-heart, and tumor-to-lung were 6.69 ± 0.91, 4.43 ± 0.61, and 3.96 ± 0.54, respectively. In addition, in the dual tumor grafts model, antibody uptake by both CD25-negative and positive tumor cells was detected within 5 h after injection. However, the retention time of Dmab(scFv)-Fc antibody in CD25-positive Hut102 tumor graft was much longer than that in CD25-negative LS174T tumor graft. Antibody uptake was only detected in Hut102 tumor graft at 7 h and 9 h after injection (Figure 5(d)). These results demonstrate that the CF750-labeled Dmab(scFv)-Fc antibody can target CD25-positive Hut102 tumor xenografts in mice.

## 4. Discussion 

In this paper, we produced a soluble Dmab(scFv)-Fc antibody against CD25 via recombinant expression in* P. pastoris*. The specific binding of this antibody to CD25-positive Hut102 tumor cells was confirmed using cultured cells and tumor tissues. In mice bearing Hut102 tumor xenografts, rapid uptake of the ^131^I-Dmab(scFv)-Fc antibody was observed within 1 h after injection, and clear imaging of the tumor xenograft could be obtained at 5 h after injection using SPECT/CT scanning. Intravenously injected CF750-Dmab(scFv)-Fc antibody rapidly accumulated in Hut102 tumors at a high level within 1 h after injection. High-contrast optical imaging of the tumor xenograft was achieved kinetically within 9 h using the CF750-Dmab(scFv)-Fc antibody. These results demonstrate that both ^131^I- and CF750-labeled Dmab(scFv)-Fc antibodies can target CD25-positive tumors, and they might be developed as imaging agents for the diagnosis of lymphomatous types of ATLL.

To be developed as a tumor imaging agent, an antibody must have the following essential characteristics: low immunogenicity, high specificity and affinity for antigen, rapid targeting and clearance, and efficient production and purification [25, 28]. Usually, humanized antibodies with low immunogenicity enable repeat dosing. The Dmab(scFv)-Fc antibody consists of the variable region of a humanized daclizumab antibody and the constant region of a human IgG1 antibody [27]. Except for the additional His-tag, the Dmab(scFv)-Fc antibody could be considered as a humanized antibody. The His-tagged Dmab(scFv)-Fc antibody was expressed in* P. pastoris* at a high level [27]. Approximately 60–70 mg His-tagged antibody was obtained by single step of Ni-NTA affinity chromatography from 1 L of culture. This efficient production and purification system is essential for the application of the Dmab(scFv)-Fc antibody for tumor imaging. Regarding the immunogenicity of the additional His-tag, the Dmab(scFv)-Fc antibody without a His-tag could also be expressed in* P. pastoris *and further purified using protein A affinity chromatography. Moreover, SDS-PAGE analysis demonstrated that incorporation of the Fc fragment made the Dmab(scFv)-Fc antibody dimeric (Figure 1(a)). The divalent Dmab(scFv)-Fc antibody is similar to the monovalent Dmab(scFv) antibody in binding specificity (Figure 2), but the Dmab(scFv)-Fc antibody displayed greater cell binding ability than the Dmab(scFv) antibody (Figure 3). In addition, the Dmab(scFv)-Fc antibody is stable at 37°C for 24 h and at 4°C for at least 1 week (data not shown).These characteristics make the Dmab(scFv)-Fc antibody an attractive candidate for use as a tumor imaging agent.

The rapid* in vivo* kinetics of the antibody is helpful for developing a time-saving imaging protocol. Usually, conventional intact antibody protocols require a 5–7 days delay between administration of antibody and image acquisition [25]. The long circulation time of intact antibodies is beneficial for their therapeutic outcome. However, when a payload such as a radionuclide is conjugated to the antibody, the long circulation time of the antibody might enhance the radioactive risk to normal tissues [25]. Thus, it is better to use engineered antibodies with a short half-life to deliver radionuclides, allowing for a next day or same-day imaging protocol. As shown in Table 1, maximal tumor uptake of the ^131^I-Dmab(scFv)-Fc antibody was detected at 1–3 h after injection, indicative of rapid targeting of the Dmab(scFv)-Fc antibody. In addition, the activity of the ^131^I-Dmab(scFv)-Fc antibody in the blood, kidney, and liver decreased from 40–50% at 1 h to 4–8% at 24 h after injection, indicating the rapid kinetics of this antibody (Table 1). Rapid clearance of the ^131^I-Dmab(scFv)-Fc antibody resulted in a rapid increase of the tumor-to-muscle ratio. Consequently, a clear image was obtained within several hours after injection (Figure 4). Clear visualization of the tumor graft was possible at 1 h after injection using the CF750-Dmab(scFv)-Fc antibody, confirming the rapid tumor uptake of Dmab(scFv)-Fc antibody (Figure 5(a)). In contrast to the long circulation time of the scFv-Fc antibody produced in mammalian cells, systemic clearance of the Dmab(scFv)-Fc antibody produced by* P. pastoris *might be accelerated by mannose glycosylation that is provided by the host cells [23, 30]. These results suggest that a time-saving imaging protocol might be developed using the Dmab(scFv)-Fc antibody produced in* P. pastoris*.

However, the Dmab(scFv)-Fc antibody might be limited by its low ratio of tumor to blood. On one hand, internalization of IL2R might shorten the tumor retention of Dmab(scFv)-Fc antibody. On the other hand, both disassociation of ^131^I and CF750 dye from antibody and degradation of labeled antibody would increase the active signal in blood and kidney. Therefore, other radioactive metals and NIR dyes should be considered in future work. In addition, the Dmab(scFv)-Fc antibody might also be limited by the activity of Fc fragment. First, the binding of the Fc fragment to FcRn would mediate antibody endocytosis and prolong its persistence in the circulation [31]. Although the mannose glycosylation provided by* P. pastoris* would reduce the circulation time of the antibody, modulation of the interaction between the Fc fragment and FcRn might further accelerate antibody clearance [23]. Second, binding of the Fc fragment to Fc receptors (FcR) on immune cells might produce antibody-dependent cell cytotoxicity [32]. In contrast to the Dmab(scFv) antibody lacking the Fc fragment, both the ^131^I- and CF750-labeled Dmab(scFv)-Fc antibodies localized to the spleen at a high level (Table 1, Figure 5(a)), suggesting the Fc fragment-mediated binding of the Dmab(scFv)-Fc antibody to splenocytes. The unexpected interaction between this antibody and immune cells might open the door for side effect and safety concerns. To modulate the binding of Dmab(scFv)-Fc antibody to FcRn and FcR, mutation at key amino acids could be introduced into its Fc fragment [32, 33]. In addition, developing a diabody and minibody lacking the Fc fragment could also be considered in the future.

Currently, radiolabeled antibodies are widely used for both clinical and experimental tumor diagnosis. However, the use of radiolabeled antibodies is limited due to their radioactive risk. The low absorption of NIR probes allows NIR light to penetrate deeper into tissue, enhancing its sensitivity. The low autofluorescence of tissue in the NIR region significantly enhances the signal-to-background ratio [34]. In this experiment, the CF750-Dmab(scFv)-Fc antibody was comparable to the ^131^I-Dmab(scFv)-Fc antibody in tumor targeting, uptake, and clearance. Compared to the radiolabeled antibody, the CF750-labeled antibody allows for kinetic visualization of the same animal bearing tumor graft. In addition, rapid progresses in optical devices, fluorochrome design, and conjugation methods will soon make NIR-based imaging a more practical and preferable alternative to radioisotope-based imaging, at least in scientific research settings [26, 35].

## Figures and Tables

**Table 1:** Tissue distribution of ^131^I-labeled Dmab(scFv)-Fc antibody in mice bearing Hut102 xenograft (*n* = 3).

	1 h	3 h	5 h	9 h	24 h
Tissue					
Blood	48.70 ± 0.91	36.95 ± 3.55	22.42 ± 0.38	14.24 ± 0.06	4.35 ± 0.25
Heart	31.15 ± 6.22	20.31 ± 4.21	10.25 ± 1.34	6.10 ± 0.75	1.53 ± 0.14
Liver	39.01 ± 8.19	28.29 ± 4.10	20.72 ± 0.84	16.87 ± 1.44	6.05 ± 1.34
Spleen	52.92 ± 7.16	31.98 ± 1.50	20.17 ± 3.61	15.61 ± 0.76	5.00 ± 2.24
Lung	32.59 ± 4.81	28.09 ± 3.91	13.92 ± 2.91	8.38 ± 1.06	1.91 ± 0.18
Kidney	47.32 ± 5.79	40.12 ± 6.90	29.85 ± 2.39	22.12 ± 1.90	8.48 ± 0.39
Brain	2.47 ± 1.59	3.50 ± 0.77	1.43 ± 0.33	0.74 ± 0.12	0.17 ± 0.06
Muscle	11.37 ± 2.97	10.43 ± 2.33	4.77 ± 1.52	3.20 ± 0.48	0.57 ± 0.09
Tumor	28.77 ± 6.43	28.94 ± 5.81	19.98 ± 5.15	13.43 ± 1.12	3.61 ± 0.37
Ratio					
Tumor/brain	8.56 ± 1.98	8.34 ± 1.39	13.9 ± 0.82	18.49 ± 4.32	22.42 ± 7.21
Tumor/muscle	2.61 ± 0.75	2.79 ± 0.38	4.33 ± 0.94	4.27 ± 0.85	6.45 ± 1.16

Tumor and normal tissue uptakes are expressed as percent injected dose per gram (%ID/g ± SD).
